# Testing continuum models of psychosis: No reduction in source monitoring ability in healthy individuals prone to auditory hallucinations

**DOI:** 10.1016/j.cortex.2016.11.011

**Published:** 2017-06

**Authors:** Jane R. Garrison, Peter Moseley, Ben Alderson-Day, David Smailes, Charles Fernyhough, Jon S. Simons

**Affiliations:** a Department of Psychology, University of Cambridge, UK; b Behavioural and Clinical Neuroscience Institute, University of Cambridge, UK; c Psychology Department, Durham University, UK; d School of Psychology, University of Central Lancashire, UK; e School of Health and Social Sciences, Leeds Trinity University, UK

**Keywords:** Schizophrenia, Auditory verbal hallucinations, Reality monitoring, Internal source monitoring

## Abstract

People with schizophrenia who hallucinate show impairments in reality monitoring (the ability to distinguish internally generated information from information obtained from external sources) compared to non-hallucinating patients and healthy individuals. While this may be explained at least in part by an increased externalizing bias, it remains unclear whether this impairment is specific to reality monitoring, or whether it also reflects a general deficit in the monitoring of self-generated information (internal source monitoring). Much interest has focused recently on continuum models of psychosis which argue that hallucination-proneness is distributed in clinical and non-clinical groups, but few studies have directly investigated reality monitoring and internal source monitoring abilities in healthy individuals with a proneness to hallucinations. Two experiments are presented here: the first (*N* = 47, with participants selected for hallucination-proneness from a larger sample of 677 adults) found no evidence of an impairment or externalizing bias on a reality monitoring task in hallucination-prone individuals; the second (*N* = 124) found no evidence of atypical performance on an internal source monitoring task in hallucination-prone individuals. The significance of these findings is reviewed in light of the clinical evidence and the implications for models of hallucination generation discussed.

## 1 Introduction

Auditory verbal hallucinations (AVH), or the experience of hearing a voice in the absence of any speaker, are experienced by a large proportion of individuals with a diagnosis of schizophrenia, as well as those with other psychiatric diagnoses such as bipolar disorder and post-traumatic stress disorder (PTSD), and by approximately 1% of the healthy population (Kråkvik et al., 2015). Cognitive and neuroscientific studies aimed at understanding the underlying mechanisms of AVH have compared task performance and/or neural activation between individuals with psychiatric diagnoses who hallucinate and those who do not (Stephane, Kuskowski, McClannahan, Surerus, & Nelson, 2010), as well as between groups of individuals with no clinical diagnoses who report differing levels of hallucination-proneness (Larøi, Van der Linden, & Marczewski, 2004). One of the most prominent cognitive models of AVH holds that these symptoms occur when internal mental events, such as inner speech, are misattributed to an external, non-self-generated source (Bentall, 1990, Frith, 1992, Moseley et al., 2013). As such, research has focused on the question of how we typically distinguish between different sources of information, and how these processes might fail.

The Source Monitoring Framework addresses how we make judgements about the origin (source) of remembered information, using characteristics such as perceptual, semantic, or affective content, or the nature of the earlier cognitive operations (Johnson, Hashtroudi, & Lindsay, 1993). Source monitoring can be broadly divided into three sub-categories depending on the contrasts which must be made: 1. External source monitoring, where the distinction is between non-self-generated sources of information, such as whether an image appeared on the left or right side of a screen; 2. Internal source monitoring, where a distinction must be made between self-generated sources of information, such as whether a sentence had previously been spoken aloud or internally using inner speech; and 3. Reality monitoring, involving discrimination between internal and external sources of information, such as whether a sentence had been spoken by the individual or by someone else, or even whether an event had been witnessed or dreamt. Each of these variants are commonly tested using a source memory paradigm, requiring the participant to encode stimuli from different sources, and on later re-presentation of the stimuli, to judge the original source of the stimuli. For example, a reality monitoring task might present participants with a series of verbal word-pairs (e.g., *bubble and squeak*), which are shown either completed (‘perceived’, that is externally generated, e.g., *bubble and squeak*) or where the second word must be supplied by the participant (‘imagined’, that is, internally generated, e.g., *bubble and s____*). Reality monitoring ability might then be assessed by asking the participant to remember whether the second word of the word-pair had previously been perceived or imagined.

Reality monitoring ability in healthy individuals is associated with activity in the medial anterior prefrontal cortex, (PFC, e.g., Simons et al., 2008, Simons et al., 2006, Turner et al., 2008), as well as to structural morphology of the nearby paracingulate sulcus (PCS; Buda, Fornito, Bergstrom, & Simons, 2011). Patients with schizophrenia show impairments in reality monitoring ability (e.g., Anselmetti et al., 2007, Brébion et al., 2000, Waters et al., 2004), which are associated with dysfunction in the medial anterior PFC (Subramaniam et al., 2012, Vinogradov et al., 2008), as well as to altered PCS morphology (Garrison, Fernyhough, McCarthy-Jones, Haggard, & Simons, 2015). Indeed, Garrison et al. (2015) indicated that a shorter PCS was associated with a higher likelihood of hallucinations in patients with schizophrenia, with these findings together suggesting that the PCS, and surrounding anterior medial PFC, may be associated with both reality monitoring and hallucinations. Considering the wider underlying network for AVH, an fMRI study with healthy individuals observed increased activation in the area surrounding the auditory cortices in the superior temporal gyrus (STG) during the encoding stage of a reality monitoring task, which correlated with measures of hallucination-proneness (Sugimori, Mitchell, Raye, Greene, & Johnson, 2014). Both the PCS and STG regions are often observed to be active during the experience of AVH in ‘symptom-capture’ fMRI studies (e.g., Zmigrod, Garrison, Carr, & Simons, 2016).

To test the suggestion that reality monitoring deficits play a causal role in the generation of AVH, research has focused on the behavioural association between atypical source monitoring and the presence or intensity of hallucinations. Two mechanisms have been proposed which might explain this deficit: an externalizing bias and a general source monitoring deficit. The idea of externalizing bias stems from the observation made during reality monitoring studies involving healthy individuals, that participants often exhibit a greater likelihood of falsely attributing new or internally generated items to an external source, than of making the reverse error (Johnson, Raye, Foley, & Foley, 1981; see Garrison, Bond, Gibbard, Johnson, & Simons, 2016, for a discussion). Bentall (1990) argued that since hallucinations are internally generated events experienced as external, atypical source monitoring in individuals with AVH is most likely to manifest itself as an enhanced externalizing bias (in which self-generated information is more likely to be misattributed as externally-generated). Behavioral evidence supports this proposal, with a recent meta-analysis finding that patients with hallucinations have a greater tendency to misattribute internal items to external sources than non-hallucinating individuals or healthy controls (Brookwell, Bentall, & Varese, 2013).

An alternative possibility is that individuals with AVH exhibit general source monitoring deficits, which can be observed in terms of poorer performance across all types of source memory tasks. Such a deficit might arise in addition to an externalizing bias (e.g., the deficit might be explained by some variation in the application of criteria used to determine the internal/external nature of mental experience), or may itself be related to the generation of the bias (e.g., if the weak application of decision-making criteria generally has a greater impact on the recognition of self-generated status than of external status). Evidence suggests that as well as deficits in reality monitoring, patients with schizophrenia do often exhibit internal and external source memory deficits when compared to healthy controls (Achim & Weiss, 2008). Furthermore, the few studies which have compared source monitoring deficits in patients with and without hallucinations offer some support for an association between general source monitoring deficits and hallucinations (Franck et al., 2000; Gawęda, Woodward, Moritz, & Kokoszka, 2013).

Interpreting the results of such empirical comparisons between patients and healthy individuals can be affected by the confound of medication status, and by other factors. Continuum models of psychosis, which suggest that that experiences such as AVH are distributed throughout the general population, infer that studying non-clinical individuals with a proneness to hallucinate can provide a useful model of clinical syndromes (van Os & Reininghaus, 2016). Based on this approach, a small number of studies have investigated whether individuals with no psychiatric diagnosis who report hallucinatory experiences exhibit the same bias and/or deficit in source monitoring that has been associated with patients with schizophrenia. This area remains under-researched – in their review, Brookwell et al. (2013) reported three source monitoring studies in non-clinical populations, only one of which has been published. Larøi et al. (2004) tested undergraduate students on a reality monitoring task, classifying participants according to their score on a self-report questionnaire, the Launay-Slade Hallucination Scale (LSHS). They found that high hallucination-prone individuals were more likely to misattribute self-generated words as having been spoken by the experimenter than those in the low hallucination-prone group, whereas there was no difference between the groups in other errors, or in recognition memory for previously presented words. However, in contrast, one study published since the Brookwell et al. meta-analysis found no effect of non-clinical hallucination-proneness on reality monitoring (Subject/Experimenter discrimination; McKague, McAnally, Skovron, Bendall, & Jackson, 2012).

It thus remains unclear whether the reality monitoring impairment observed in patients with schizophrenia who hallucinate is also present in non-clinical hallucination-prone samples. Furthermore, to our knowledge, no study has tested the relationship between hallucination-proneness in a non-clinical sample and performance on internal source monitoring tasks, which might support the presence of a generalised deficit in source monitoring in the generation of hallucinations. Here, we report data from two separate experiments conducted with individuals recruited from two university populations, which investigated whether non-clinical hallucination-proneness is associated with impairments in source memory performance, and if so, whether this is explained by an externalizing bias and/or a general internal source monitoring deficit. Experiment 1 recruited participants who scored in the top or bottom quartiles of a version of the LSHS, and tested for an association between self-reported hallucination-proneness and reality monitoring performance. Experiment 2 tested for an association between hallucination-proneness and internal source monitoring performance (overt/covert speech judgements). The externalizing bias model of AVH would predict that, on the reality monitoring task, higher hallucination-proneness should be associated with a greater tendency towards incorrectly responding that words spoken by the participant were spoken by the experimenter, and that word-pairs which had been imagined should be judged to have been perceived. If such effects reflect an externalizing bias, they should be specific to the reality monitoring task, with no difference observed on the internal source monitoring task. Alternatively, a general source monitoring deficit account of AVH would predict that higher hallucination-proneness would be associated with lower overall performance on both the reality monitoring task and the internal source monitoring task.

## 2 Experiment 1: reality monitoring

### 2.1 Methods

#### 2.1.1 Participants

677 participants were recruited to an on-line questionnaire by email invitation from volunteer lists maintained at the Behavioural and Clinical Neuroscience Institute at Cambridge University, and the Department of Psychology, Durham University, and from advertisements in the Cambridge and Durham areas. There was no financial incentive to participate and ethical approval for the study was obtained from the University of Cambridge Psychology Research Ethics Committee. An individual's proneness to auditory hallucinations was assessed using a modified version (Morrison, Wells, & Nothard, 2000) of the Predisposition to Auditory Hallucination subscale of the Revised Launay-Slade Hallucination Scale (LSHS-R, Bentall & Slade, 1985; see Section 2.1.2). Individuals who had LSHS-R scores in the upper or lower quartile (High-LS, or Low-LS) indicating high or low proneness to auditory hallucinations were invited for testing using the reality monitoring task in the Department of Psychology at either the University of Cambridge or Durham University.

Twenty-five individuals were tested in the High-LS group (number of females = 18; mean age = 19.8, SD = 2.8 years; mean LSHS-R score = 13.2, SD = 2.1), and 22 individuals in the Low-LS group (number of females = 20; mean age = 22.9, SD = 7.5 years; mean LSHS-R score = 2.1, SD = 1.4). Proneness to auditory hallucinations, as measured by the LSHS-R, differed significantly between these groups: *t*(45) = 20.973, *p* < .001. There were no significant differences between the groups in terms of age [*t*(45) = 1.932, n.s.] or sex (*χ*^2^ = 2.703, n.s.), all participants reported being native English speakers, and no participants reported any hearing problems.

#### 2.1.2 Design and procedure

Self-report measures – The revised version of the Launay-Slade Hallucination Scale (LSHS-R), used to assess predisposition to hallucinatory experiences in the auditory modality, comprises five questions (e.g., *I have had the experience of hearing a person's voice and then found that no-one was there*), with each item scored on a five-point Likert scale ranging from ‘never’ (0) to ‘almost always’ (4). Total scores can thus range from 0 to 20 with higher scores indicating a greater predisposition to auditory hallucinations. The original scale was modified by McCarthy-Jones and Fernyhough (2011) to remove a question with a low endorsement rate and improve internal reliability, and in testing was found to have satisfactory psychometric properties.

**Fig. 1:**
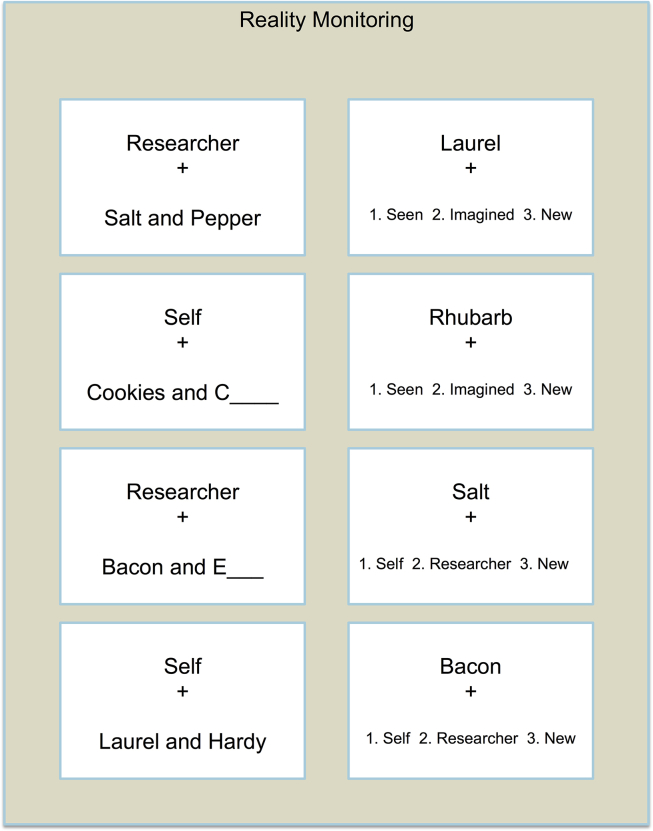
**Stimuli used in the Reality Monitoring Tasks**. Note: Sample stimuli used in the study phase (left) and test phase (right) of the reality monitoring task. In a 2 × 2 design, either the subject or experimenter spoke aloud the stimuli, which were presented either complete (perceived) or incomplete (requiring the second word to be imagined). Subjects were then presented at test with the first word of a word pair, and asked to judge whether the accompanying word had been seen or imagined, or if the presented word was new; or whether the subject or experimenter had read aloud the word pair, or the presented word was new.

Reality monitoring task – The task was adapted from one used previously (Simons et al., 2006, Simons et al., 2008) and involved the initial presentation of word-pairs followed by a test phase. In the test phase, the participant was asked to indicate whether a word had earlier been presented within an intact word-pair using the response ‘Perceived’, or had been presented in a word-pair which had needed to be completed by imagining the missing word, with the response ‘Imagined’. Participants were also required to judge whether the word-pair had previously been spoken aloud by themselves (‘Subject’ response) or was spoken by the researcher (‘Experimenter’ response). Previously unstudied words were also used in the test phase, requiring a ‘New’ response. The stimuli consisted of 216 well-known word-pairs (e.g., ‘*Laurel and Hardy’*, ‘*Bacon and Eggs’*), which were pilot tested for the current study to ensure their familiarity among adults in the target demographic range. The task comprised 6 separate study and test blocks, with 24 word-pair stimuli in each study phase (six word-pairs presented in four combinations of Subject/Experimenter × Perceived/Imagined conditions; Fig. 1) and an additional 12 new words included in the test phase.

Each study trial commenced with a screen indicating whether the subject or experimenter should read aloud the word-pair. The word-pair was then shown, either complete (perceived trials) or with only the first letter of the second word provided such that the second word needed to be self-generated (imagined trials). In both cases the subject or experimenter then had 3 s to read aloud the entire word-pair, completing the word-pair as necessary for imagined trials. Each study phase was followed by its corresponding test phase, consisting of one sub-block for each of the two reality monitoring conditions. The sub-blocks commenced with a question screen indicating which condition was being tested, i.e., for the Perceived/Imagined condition: ‘*Was the accompanying word Seen or Imagined or New?*’, and for the Subject/Experimenter condition: ‘*Was the accompanying word said by Self or Researcher or New*?’ These were then followed by a test screen containing the first word from one of the studied word-pairs, or a new word, together with the instruction to provide the appropriate response. Participants had 4 sec to make their response.

The order of presentation of sub-blocks in the test phase alternated across the six full blocks of the task and was counterbalanced across participants. The word-pairs assigned to the Perceived/Imagined and Subject/Experimenter conditions, as well as new words, were also counterbalanced across participants, and the order of presentation of word-pairs was pseudo-randomized to ensure no run of more than three items of the same condition in any study or test phase.

#### 2.1.3 Data analysis

Old/New recognition accuracy was calculated as the adjusted item recognition score (hits minus false alarms, with hits being defined as the proportion of words correctly recognised as previously seen and false alarms the proportion of new words incorrectly endorsed as old). Reality monitoring accuracy was calculated as the number of accurate source responses divided by the number of correct responses recognising an item as old.

Misattribution errors were calculated for perceived and imagined trials as the number of responses made for the alternative reality monitoring response as a proportion of total errors made. So for example, ‘Imagined judged Perceived’ errors were calculated as the number of perceived responses that were made to imagined trials divided by the sum of perceived and new responses to imagined trials. This gives a measure of misattribution error unrelated to overall item recognition accuracy for each condition. The proportion of internalisation errors (Perceived judged Imagined, or Experimenter judged Subject) was then compared to the proportion of externalisation errors (Imagined judged Perceived or Subject judged Experimenter) for each participant, to give a measure of externalizing bias. Eight participants made no errors for one or more of the study conditions for the Perceived/Imagined task (6 in the High-LS and 2 in the Low-LS conditions) and 15 (8 in the High-LS and 7 in the Low-LS conditions) for the Subject/Experimenter task; these participants were excluded from the misattribution bias analysis only.

Preliminary analyses confirmed the absence of significant effects of potentially confounding variables of participants' age or sex on Old/New recognition, reality monitoring accuracy or externalizing bias, all *F*'s(1,44) < 3.291, n.s.

### 2.2 Results

There was no difference between the high and low hallucination proneness groups for Old/New memory, *t*(45) = .416, *p* = .679, *d* = .115, indicating that the groups had similar recognition memory ability (Table 1).

**Table 1:** Old/new recognition and reality monitoring accuracy.

Accuracy variable	Low-LS	High-LS	t statistic (df = 45)	*p*
	*M* (*SD*)	*M* (*SD*)		
**Old/New recognition**	**.85 (.11)**	**.86 (.05)**	**−.416**	**.68**
**Perceived/Imagined reality monitoring**	**.85 (.07)**	**.84 (.08)**	**.385**	**.70**
**Subject/Experimenter reality monitoring**	**.92 (.05)**	**.93 (.05)**	**−.319**	**.75**
Subject/Experimenter: Subject generated	.88 (.08)	.90 (.08)	−.583	.56
Subject/Experimenter: Experimenter generated	.96 (.04)	.96 (.03)	.509	.62

Note: To aid comparison with the findings of Larøi et al. (2004) the results of the Subject/Experimenter reality monitoring task were further broken down for trials which had been spoken by the subject or experimenter (results shown in un-bolded text).

To analyse the reality monitoring data, a mixed ANOVA with High-LS and Low-LS group as between-subjects factor, and the reality monitoring condition (Subject/Experimenter or Perceived/Imagined) as a within-subject factor, was conducted. There was a within subjects effect of task condition: *F*(1,45) = 64.479, *p* < .001, η_p_^2^ = .589, indicating that both groups were better at judging whether a word-pair had been spoken by the subject or experimenter, compared to distinguishing whether a word-pair had been perceived or imagined. However, there was no main effect of hallucination-proneness group, *F*(1,45) = .014, *p* = .905, η_p_^2^ = .000, and no interaction between hallucination-proneness group and reality monitoring condition: *F*(1,45) = .460, *p* = .501, η_p_^2^ = .010, thus giving no indication of an association between hallucination proneness and reality monitoring ability.

To allow a direct comparison with the findings of the similar study by Larøi et al. (2004), the results of the Subject/Experimenter reality monitoring task were then broken down for trials which had been spoken by the subject or by the experimenter (Table 1). Contrary to the findings of the earlier study, a mixed ANOVA with Subject/Experimenter accuracy as DV, group as factor and whether the word-pair had been spoken by the subject or experimenter (‘speaker’) as within-subjects variable, revealed that while both groups were better at the Subject/Experimenter discrimination for word-pairs spoken by the Experimenter, *F*(1, 45) = 31.744, *p* < .001, η_p_^2^ = .414, there was no group difference in subjects' ability to remember that they had previously spoken the word-pair, compared with their memory for experimenter spoken stimuli, i.e., no significant group × speaker interaction: *F*(1, 45) = .649, *p* = .425, η_p_^2^ = .014.

**Fig. 2:**
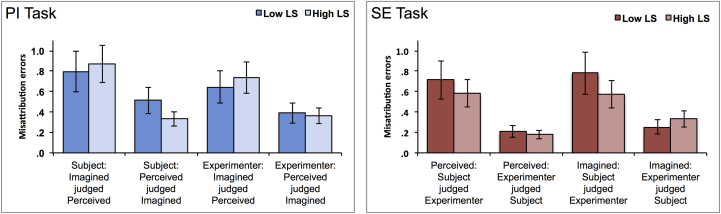
**Misattribution errors**. Note: The two charts refer to items misclassified for each of the reality monitoring tasks, broken down by the trial conditions. So for example, the first 2 bars in the left chart refer to items which had been imagined by the subject, which were then incorrectly judged as perceived (an externalisation error), and the last 2 bars in the right chart to items which had been imagined by the experimenter during encoding, but which the subject had later judged to have been self-imagined (an internalisation error).

Finally, an analysis of errors was undertaken by calculating a misattribution error rate as a measure independent of reality monitoring accuracy, to give an indication of the proportion of errors that were ascribed to the alternate reality monitoring condition as opposed to a new item (Fig. 2). A mixed ANOVA was undertaken for the analysis of errors on the Perceived/Imagined task, with the misattribution error rate as DV, group as factor, and two within-subject variables of whether the trials has been spoken by subject or experimenter, and whether the error direction was internalising (that is, Perceived judged Imagined) or externalising (that is, Imagined judged Perceived). The analysis revealed no significant group difference for the proportion of misattribution errors made overall, *F*(1, 37) = .051, *p* = .823, η_p_^2^ = .001, but with a consistent externalizing bias for both groups, as measured by a greater number of externalisation compared to internalisation errors for each condition: *F*(1, 37) = 59.146, *p* < .001, η_p_^2^ = .615. This externalising bias was not significantly different for items which had been spoken by the subject or the experimenter: *F*(1,37) = 1.276, *p* = .266, η_p_^2^ = .033.

The analysis of variance of misattribution errors in the Perceived/Imagined tasks did reveal a marginal interaction between group and internal–external error direction, *F*(1, 37) = 3.838, *p* = .058, η_p_^2^ = .094, suggesting that participants in the High-LS group might have some tendency to make more externalizing errors than participants in the Low-LS group. However, when this possibility was tested, there was found to be no overall difference between the groups in either the proportion of externalising errors, [I judged P: t(45) = −.995, *p* = .326, *d* = .291], or internalising errors, [P judged I: t(45) = .439, *p* = .663, *d* = .127]. Furthermore, the absence of a three way, group × error direction × (spoken by subject or experimenter) condition interaction, *F*(1, 37) = .654, *p* = .424, η_p_^2^ = .017, suggests that any such tendency was not associated with information that had been specifically generated by the subject, as opposed to by the experimenter.

A similar analysis of variance undertaken for the Subject/Experimenter task also revealed an externalizing bias for both groups: *F*(1, 30) = 42.594, *p* < .001, η_p_^2^ = .587, suggesting that participants were more likely to ascribe a word-pair spoken by themselves to the experimenter than they were a word-pair spoken by the experimenter to themselves. This was the case regardless of whether the stimulus had earlier been perceived or imagined at encoding: *F*(1, 30) = .274, *p* = .605, η_p_^2^ = .009. There was no difference between the groups for the proportion of misattribution errors made, nor any other significant main effects or interactions, *F*(1,30) < 2.231, *p* > .146, η_p_^2^ < .069.

The analysis of misattribution errors across both reality monitoring conditions therefore gives no evidence of an enhanced externalising bias in individuals with a greater proneness to AVH.

## 3 Experiment 2: internal source monitoring

The second experiment used an internal source monitoring paradigm, requiring participants to either read a word-pair to themselves using inner speech (i.e., covert speech), or to read a word-pair aloud (overt speech). At a later point, participants were presented with each of the word-pairs again, and were required to recall whether each had been read silently or aloud. Given that this task required participants to make a decision between only two options (covert or overt speech) about each word-pair, the data was analyzed using signal detection theory to investigate both sensitivity and bias on the task. It was hypothesized that, if hallucination-proneness is associated with a general source monitoring deficit, there should be a significant positive correlation between LSHS-R score and internal source monitoring task performance (task sensitivity).

### 3.1 Method

#### 3.1.1 Participants

The sample consisted of 125 participants from the staff and student population of Durham University, UK. One participant was excluded from further analysis because their task sensitivity (*d'*) on the source monitoring task was <0, indicating below chance performance, leaving a final sample size of 124 (number of females = 96, mean age = 20.7 years, SD = 2.5 years). Participants all reported being native English speakers, and no participants reported any hearing problems. Mean score on the LSHS-R was 8.75 (SD = 2.11). When participants were categorized into high and low hallucination-proneness groups, there was no difference in age or sex between groups [age: *t*(42) = 1.32, *p* = .195; sex: *χ*^2^ = 1.91, *p* = .167]. The high hallucination-prone group scored significantly higher on the LSHS-R (*M* = 11.85, SD = .93) than the low hallucination-prone group (*M* = 5.61, SD = .61); *t*(42) = 25.06, *p* < .001.

#### 3.1.2 Design and procedure

The auditory items from the revised version of the LSHS-R were again used to assess hallucination-proneness (see Section 2.1.2), although in this experiment response options ranged from 1 to 4 for each question, with total scores thus able to range from 4 to 20, compared with 0–20 in Experiment 1. This difference arose due to an error in the reporting of previous literature; McCarthy-Jones and Fernyhough (2011) described their revised version of the LSHS as comprising questions with response options 0–4, which was the basis for the questionnaire used in Experiment 1. However, in a later corrigendum, McCarthy-Jones and Fernyhough clarified that a 1–4 scale had actually been used, and this corrected version was adopted for our Experiment 2. Therefore, although the questionnaires used in our two experiments consisted of exactly the same questions, the mean scores are not directly comparable.

Internal source monitoring task **–** In contrast to the task used in Experiment 1, this source memory task, based loosely on that used by Franck et al. (2000), asked participants to distinguish between two internally generated sources: whether verbal stimuli were spoken aloud using overt speech, or said silently to themselves using covert (inner) speech. As with the reality monitoring task, there were two stages to the task, involving word-pair completion and subsequent recall. Participants were not informed that they would be asked to remember the word-pairs until immediately before the second stage of the task.

In the word-pair completion stage, participants were presented with a series of 72 word-pairs (for example, ‘*gold and silver’*), 36 of which they were instructed to say out loud (‘overt speech’ trials), and 36 of which they were instructed to say to themselves using inner speech (‘covert speech’ trials). To manipulate the extent to which the stimuli were self-generated, and in a similar way to the reality monitoring task in Experiment 1, within each condition, 18 word-pairs were fully presented to participants on-screen (e.g., ‘*gold and silver’*, viz. ‘perceived’ trials), while the remaining 18 were only partially completed (e.g., ‘*gold and s_____*’, viz. ‘imagined’ trials). For each trial, the participant was asked to say the full word-pair (overtly or covertly); thus, half of the trials required participants to produce the words themselves (imagined), whereas half required the participant to read the word-pair from the screen (perceived). The word-pairs were informally tested in a small pilot study, to ensure that they were familiar to the large majority of participants. Word-pairs were counterbalanced across presentation mode (perceived or imagined) and condition (overt/covert). For each trial, the instruction (‘Out Loud’ or ‘Inner Speech’) appeared on the screen for 1250 msec, followed by the word-pair for 3250 msec, followed by an inter-trial interval of 750 msec. If the participant did not know the correct word to complete a pair, they were instructed to indicate this with a button press.

After the first stage was completed, participants took a 15 min break, during which they completed the LSHS-R, as well as various other self-report questionnaires relating to inner speech phenomenology (Varieties of Inner Speech Questionnaire; McCarthy-Jones & Fernyhough, 2011), and anxiety and depression (Hospital Anxiety and Depression Scale; Zigmond & Snaith, 1983). These measures were not intended to be linked to the source memory task, but were included in association with other task-based measures that were completed later in the testing session (to be reported elsewhere; Moseley et al., in preparation). After 15 min, participants were then asked to complete the memory test stage of the task, in which they were presented with the second part of each word-pair (e.g., ‘*silver’*), in a random order. Participants were asked to try to judge whether they had previously said each word out loud or using inner speech, responding with a button press. This test phase was self-timed with each word presented in the centre of the computer screen until a response was made.

#### 3.1.3 Data analysis

Signal detection measures were used to analyze data from the internal source monitoring task, as recommended by McKague et al. (2012). ‘Hits’ were classified as overtly spoken words correctly recalled as such, whilst false alarms were classified as covertly spoken words incorrectly recalled as overtly spoken (‘Miss’ and ‘correct rejection’ responses are not reported here, since they are, necessarily, directly proportional to hit and false alarm rates). *d'*, a measure of task sensitivity, was calculated as the standardised hit rate (*z*-score of hit rate) minus the standardised false alarm rate (*z*-score of false alarm rate), with a lower value indicating less ability to distinguish the source of words. The second dependent variable was *β*, a measure of response bias, which was calculated as outlined by Stanislaw and Todorov (1999) (with a lower value indicating a lower criterion for deciding that a word was spoken aloud).

In contrast to Experiment 1, where an initial group split on hallucination-proneness was used to invite participants for behavioural testing, all participants in Experiment 2 completed both the LSHS-R self-report questionnaire and the internal source monitoring task. Therefore, we first computed correlations (Spearman's rho) between proneness to auditory hallucinations and internal source monitoring performance (sensitivity and response bias, for the imagined and perceived conditions). To enable comparison with Experiment 1, we also split participants into high hallucination-proneness (those scoring in the upper quartile on the LSHS-R; *N* = 26) and low hallucination-proneness (scoring in the lower quartile on the LSHS-R; *N* = 18) groups, and compared performance on the source monitoring task between these groups. There was a significant difference between the high and low groups in hallucination-proneness: *t*(42) = 25.06, *p* < .001, as expected. A 2 × 2 mixed model ANOVA with a between-subjects variable of hallucination-proneness group (high/low) and a within-subjects variable of task condition (imagined/perceived word-pairs) was therefore conducted with both *d'* and *β* as dependent variables.

### 3.2 Results

There were no significant correlations between proneness to auditory hallucinations and internal source monitoring performance for either of the conditions (perceived or imagined) of the internal source monitoring task, or for task performance overall, Spearman's rho ≤ .124, all *p*s > .167 (see Table 2).

**Table 2:** Correlations between internal source monitoring task performance and auditory hallucination-proneness.

SMT measure	*Spearman's rho*
*d'* (overall)	−.114
*β* (overall)	.001
*d'* (imagined	−.076
*β* (imagined)	.010
*d'* (perceived)	−.124
*β* (perceived)	−.001

Note: *d'* = task sensitivity; *β* = task response bias. Higher *d'* measures correspond to greater ability to distinguish between overtly and covertly spoken words. Higher *β* values correspond to a more conservative criterion for deciding a word was spoken overtly. None of the correlations were significant at *p* < .05, even before correction for multiple comparisons.

A 2 × 2 mixed model ANOVA with task sensitivity (*d'*) as the dependent variable (Table 3) showed a main effect of imagined/perceived status: *F*(1, 42) = 44.27, *p* < .001, η_p_^2^ = .513; sensitivity was greater for imagined word-pairs (*M* = 1.68, SD = .74) compared with those that had been perceived (*M* = 1.04, SD = .61). There was a marginal main effect of hallucination-proneness: *F*(1, 42) = 3.36, *p* = .074, η_p_^2^ = .074. There was no significant interaction between task condition (Perceived/Imagined) and hallucination-proneness (high/low): *F*(1, 42) = .03, *p* = .862, η_p_^2^ = .001.

**Table 3:** Group performance on the internal source monitoring task.

Source monitoring measure	Hallucination-proneness	
	*Low*	*High*
*d'* (imagined)	1.88 (.69)	1.61 (.66)
*d'* (perceived)	1.16 (.67)	.85 (.53)
*β* (imagined)	1.58 (1.14)	1.62 (1.36)
*β* (perceived)	1.93 (1.32)	1.83 (1.08)

Note: *d'* = task sensitivity; *β* = task response bias. Measures shown are mean scores, with SD in parentheses.

There was no difference in *β* between the task conditions, *F*(1, 42) = 1.10, *p* = .299, η_p_^2^ = .026, or hallucination-proneness groups, *F*(1, 42) = .017, *p* = .896, η_p_^2^ < .001, and no significant interaction between task condition (Perceived/Imagined) and hallucination-proneness (high/low): *F*(1, 42) = .073, *p* = .788, η_p_^2^ = .002. Thus, the experiment indicated no significant differences between hallucination-proneness groups on any measure of internal source monitoring.

## 4 General discussion

The two experiments presented above addressed a key prediction of continuum models of psychosis: whether source monitoring impairments are associated with hallucination-proneness in the non-clinical population, as they are in people with clinical diagnoses who hallucinate. Experiment 1 found no difference in accuracy either for Old/New recognition, or for Perceived/Imagined or Subject/Experimenter reality monitoring judgments, with effect sizes so low (η_p_^2^ ≤ .02) as to preclude a sample size explanation. While there was clear evidence of a general externalizing bias in both reality monitoring conditions, consistent with that previously reported from studies in the healthy population (Johnson et al., 1981), this was not found to be significantly enhanced in participants in the high hallucination-proneness group.

A marginal interaction (*p* = .058) between group and internal–external error direction in the Perceived/Imagined reality monitoring task suggested that there might be a tendency for high hallucination-prone individuals to judge a greater proportion of imagined word-pairs as perceived than perceived words as imagined. However, this possibility was not supported by analysis of the simple effects, and if such a tendency is related to auditory hallucinations, then it might be expected to be specific to items that were generated by the subject, which was not found to be the case. Furthermore, there was no evidence of an enhanced externalizing bias in the Subject/Experimenter reality monitoring task for the high hallucination-prone group compared to the low hallucination-prone group.

The results from Experiment 1 were supported by the findings of Experiment 2, which investigated participants' ability to discriminate between whether they had overtly or covertly spoken a word-pair, which had been either perceived or imagined during the encoding phase. No significant correlations were found between task sensitivity or response bias and hallucination-proneness, regardless of whether the stimuli had previously been perceived or imagined. Furthermore, no differences were detected in task sensitivity and response bias between groups of participants split by hallucination-proneness as in Experiment 1. There was a marginal reduction in overall task sensitivity for the higher hallucination-proneness group (*p* = .074), but this was not supported by a significant correlation between task sensitivity and hallucination-proneness score across the entire sample. Indeed, the correlation effect sizes were so small (rho ≤ .124) that statistical power is again unlikely to be an explanation, and there was no interaction for task sensitivity between LSHS-R group and the perceived or imagined status of the stimuli, as might be expected if the deficit related to the inability to distinguish the source of imagined information from that which had been perceived.

The results of these two experiments thus offer little or no support for a deficit in source monitoring ability, and/or of enhanced externalizing biases in hallucination-prone individuals in the healthy population. These findings contrast with the findings of behavioral and neuroimaging studies in patients with schizophrenia, which report associations between reality monitoring impairment and the presence of AVH. Indeed we have demonstrated reality monitoring impairment and dysfunction in the medial anterior PFC in patients with schizophrenia using a very similar version of the task to that used in Experiment 1 (Garrison et al., in revision). As such, the results are inconsistent with continuum models of psychosis (van Os & Reininghaus, 2016), which assert that hallucinations are distributed throughout the general population, and thus which predict comparable effects in healthy individuals who are prone to hallucinations as in patients with schizophrenia who hallucinate. However, there are a number of alternative possible explanations for the apparent discrepancy in findings relating to the association between source monitoring impairment and AVH in clinical and non-clinical groups.

Firstly, it is possible that the assessment of hallucination-proneness used in the present experiments was ineffective in measuring individuals' proneness to AVH in the non-clinical population. However, while the LSHS-R comprises only five questions which ask about unusual auditory experiences, the scale in its revised form has been well tested and found to have satisfactory psychometric properties (see McCarthy-Jones & Fernyhough, 2011).

Alternatively, there may be only some shared overlap of the mechanisms involved in clinical and non-clinical hallucinatory experiences (as suggested by Badcock & Hugdahl, 2012), which might be especially true for the hallucinatory experiences reported by the samples employed here. Larøi (2012) proposed a (fuzzy) distinction between participants in studies of non-clinical hallucinations research, referring to these as Type i non-patients and Type ii non-patients. Participants recruited in the current two experiments would be classed as Type i non-patients, who typically report infrequent hallucinatory experiences that may be phenomenologically distinct from the AVH reported by patients (e.g., brief experiences that rarely take the form of complex utterances). In contrast, Type ii non-patients often report relatively frequent hallucinations that are phenomenologically more similar to the AVH reported by patients, except in terms of emotional valence and perceived controllability (Johns et al., 2014). Thus, a reality monitoring impairment may not be involved in the hallucinatory experiences reported by Type i non-patients, but may be involved in the ‘full blown’ AVH reported by Type ii non-patients as well as by patients.

A further question relates to how reality monitoring impairment might be implicated in the generation of hallucinations. Reality monitoring is defined as a mnemonic ability, but the cognitive operations involved in monitoring the origin of retrieved information might overlap with those that monitor the origin of real time information (Johnson & Raye, 1981; discussed in; Garrison et al., 2016), with impairments in these operations leading to the generation of hallucinations. However, other mechanisms have been proposed to explain the failure to correctly identify the origin of self-generated information, which might manifest differently across different groups of individuals. For example, AVH may arise from enhanced perceptual content of self-generated auditory information due to over-activation of secondary association speech and language cortices, such as the voice-selective auditory regions in the STG (Allen, Larøi, McGuire, & Aleman, 2008). If activation of these brain areas results in unusually vivid internal auditory imagery, such information could be erroneously recognised as external in origin, without any deficit in normal source monitoring activity. Consistent with this suggestion, speech and language areas are active in addition to anterior regions such as cingulate cortices during hallucinations (Zmigrod et al., 2016), and a neuroimaging study in healthy individuals indicated the presence of intermittent episodes of significantly increased activity in bilateral primary and secondary auditory cortices, together with associated activations in the anterior cingulate cortex, even during periods of silence (Hunter et al., 2006).

Alternatively, there may be a distinct impairment in the self-monitoring processes which predict the sensory consequences of actions through comparator forward modelling/efference copy mechanisms (Feinberg, 1978). Self-monitoring accounts of reality testing suggest that AVH arise from a disruption in the capacity to monitor the intention to produce inner speech (or other cognitions), resulting in it being erroneously marked as external (Seal, Aleman, & McGuire, 2004). Such accounts thus provide an explanation for the external directionality of errors without the need for any deficit in a separate source monitoring process, as information is assumed to be externally perceived in the absence of an efference copy signal. However, while there is strong evidence for self-recognition deficits in patients with schizophrenia relating to motor action (Blakemore et al., 2002, Frith et al., 2000), and some support for corollary discharge dysfunction in schizophrenia (Ford and Mathalon, 2004, Ford et al., 2001), direct evidence for a specific comparator model relating to inner/covert speech or auditory imagery is lacking. Furthermore, theoretical arguments have been raised against the idea that the generation of thought has the same physiological consequences as that of motor action, with Gallagher (2004) arguing that the self-monitoring account of hallucinations fails in applying an explanation of motor function to one of cognitive experience.

Finally, it should be noted that the findings of the reality monitoring study in Experiment 1 are in contrast to those of Larøi et al. (2004), who reported significant differences between low and high hallucination-prone healthy individuals in the accuracy of self-generated, but not experimenter-generated, stimuli. The discrepancy appears not to be explained by the use of non-parametric statistics to analyze the results in the Larøi et al. study, as similar non-parametric analysis of our experiments did not alter our findings. What is clear, however, is that the investigation of reality monitoring and source monitoring deficits in clinical studies has also produced a range of varying results (see Brookwell et al., 2013). These might be explained by a wide variation in task design, with some tasks using verbal stimuli and others using performed or perceived actions, and with some tasks using only the Subject/Experimenter or the Perceived/Imagined discriminations separately. This may be the explanation for the discrepancy in findings with the Larøi et al. study, which used a Subject/Experimenter paradigm but with stimuli varying in emotional valence and cognitive effort. Furthermore, Larøi et al. used a version of the LSHS consisting of 16 questions, many of which seem only indirectly related to hallucination-proneness (e.g., “*I have had a sensation of floating or falling, or that I left my body temporarily*”). In contrast, the present study focused solely on auditory hallucinations. Using the same task across clinical and non-clinical groups, together with tighter control of confounding variables such as participants' age, language skills or the presence of general memory deficits, should help address variation across studies going forward.

We remain a long way from understanding the brain mechanisms underpinning hallucinations, with many existing theoretical models of AVH failing to address the complexity and diversity of hallucinations (for example, their differing developmental trajectories, or complex interactions with the individual). Understanding whether a single theoretical model can be applied to clinical and non-clinical hallucinations will depend on the flexibility of the framework to account for how factors implicated in the generation of these perceptual anomalies might interact to explain differences in phenomenological experience between groups, as well as the variety in experience for a single individual. If such a framework can be developed, this would map most closely to quasi-dimensional models of schizotypy (Yung et al., 2005), which allow for discontinuities in the experience of psychosis across the population consistent with separable phenotypic expressions of associated factors (Meehl, 1989, Meehl, 1962), rather than a fully-dimensional model (Claridge, 1972, Claridge, 1994) more supportive of an unbroken continuum. Although the work in the present study does not support the role of reality-monitoring ability as a factor in non-clinical hallucination-proneness, this does not rule out more complex models invoking reality monitoring as an important factor in the transition between hallucination-proneness and more frequent hallucinatory experiences, in clinical or non-clinical populations.
